# Quality Control of Danggui Buxue Tang, a Traditional Chinese Medicine Decoction, by ^1^H-NMR Metabolic Profiling

**DOI:** 10.1155/2014/567893

**Published:** 2014-03-23

**Authors:** Pui Hei Chan, Wendy L. Zhang, Chi Yuen Cheung, Karl W. K. Tsim, Henry Lam

**Affiliations:** ^1^Department of Chemical and Biomolecular Engineering, The Hong Kong University of Science and Technology, Clear Water Bay, Hong Kong; ^2^Divison of Life Science and Centre for Chinese Medicine, The Hong Kong University of Science and Technology, Clear Water Bay, Hong Kong; ^3^Division of Biomedical Engineering, The Hong Kong University of Science and Technology, Clear Water Bay, Hong Kong

## Abstract

Danggui Buxue Tang (DBT) is one of the simplest traditional Chinese medicine (TCM) decoctions, first described in China in 1247 AD. DBT is composed of 2 herbs, Astragali Radix (AR) and Angelica Sinensis Radix (ASR), boiled together in a 5 : 1 ratio. Clinically, DBT is prescribed to women as a remedy for menopausal symptoms. Here, H-NMR metabolic profiling was conducted for DBT and the water extracts of AR or ASR, to evaluate the potential of this chemical profiling method for quality control of the herbal decoction. Principal component analysis (PCA) showed that DBT could be readily distinguished from the water extracts of its constituent herbs by the metabolic profiles. More interestingly, the metabolic profile of DBT was not a simple sum of that of AR and ASR. Asparagine was found at significantly higher concentration in DBT than that in either AR or ASR extract, contributing mainly to the discrimination of DBT sample. In addition, we employed the same method to profile a commercial DBT powder, verifying its authenticity as compared to our prepared DBT. This study is the first to employ H-NMR metabolic profiling for the quality control of traditional Chinese medicine decoctions.

## 1. Introduction

Traditional Chinese medicines (TCMs) have been used as medicines or health supplements for more than 2500 years. TCMs are often prepared as decoctions with specific combinations of individual herbs in well-defined formulae. Among thousands of herbal formulae, Danggui Buxue Tang (DBT) is one of the simplest, composed of two herbs only. DBT was firstly described in* Neiwaishang Bianhuo Lun* by Li Dongyuan in China in 1247 AD. The DBT formula should include 10 qian (~37.3 g) of Astragali Radix (AR), roots of* Astragalus membranaceus* (Fisch.) Bunge or* Astragalus membranaceus* (Fisch.) Bunge var.* mongholicus* (Bunge) P.K. Hsiao, and two qian (~7.5 g) of Angelica Sinensis Radix (ASR), roots of* Angelica sinensis* (Oliv.) Diels. The mixed herbs should be boiled in two bowls of water (~500 mL) over a moderate heat until the final volume is reduced by half [1, 2]. Clinically, DBT is prescribed to women in China as a remedy for menopausal symptoms, in order to improve their “Qi” (vital energy) and nourish their “Blood” (body circulation).

The optimized conditions for DBT extraction were established previously [1]. By examining both chemical and biological responses, Song et al. reported that a 1 : 8 (w : v) of herb : water ratio at 2 hours of extraction time (repeating once), with both herbs boiled together rather than separately, was the best extraction method for DBT. In addition, the weight ratio of 5 : 1 (AR : ASR) produces the best DBT [3, 4]. To study the chemical composition of DBT, Yi et al. (2007) employed HPLC-DAD/ELSD and LC-ESI-MS successfully to profile 18 markers of AR and ASR in DBT [5]. Another nonprofiling study was performed by Zhang et al. (2012) to compare three different ancient formulae for DBT [6]. Their results showed that DBT made by AR : ASR in 5 : 1 ratio has the highest amount of AR-derived astragaloside III, astragaloside IV, calycosin, and formononetin, as well as ASR-derived ferulic acid: this herbal decoction has stronger activities in osteogenic, estrogenic, and erythropoietic effects than that generated from different herb ratios [6]. Thus far, the chemical analysis of DBT has focused on specific markers, in particular ferulic acid and Z-ligustilide from ASR and calycosin and formononetin and astragaloside IV from AR.

Recently, ^1^H-NMR metabolic profiling has received interest as a quick and generic way for quality control of Chinese medicinal herbs. Quality control of Ginseng Radix and ASR by using ^1^H-NMR metabolic profiling had been reported [7, 8]. However, there is so far no report on using this method to profile herbal decoctions of multiple herbs. Here, we would like to answer the question of whether it is sufficient to maintain standards for individual herbs and make use of the additive property of ^1^H-NMR to generate the expected profiles of herbal decoctions, which subsequently could be used for the purpose of quality control of such mixtures. Therefore, we performed ^1^H-NMR metabolic profiling on DBT, as well as on the water extracts of the two individual herbs AR and ASR. In addition, we tested a commercial DBT product using the same technique to demonstrate that our method can potentially be used to assess the quality of ready-to-consume TCM products on the market.

## 2. Materials and Methods

### 2.1. Materials and Reagents

Three-year-old* Astragalus membranaceus* (Fisch.) Bunge var.* mongholicus* (Bunge) Hsiao roots (AR) from Shanxi Province [9] and two-year-old* Angelica sinensis *(Oliv.) Diels roots (ASR) from Minxian in Gansu Province, China [10], were collected. The authentication of plant materials was performed morphologically by Dr. Tina Dong, during the field collection. The corresponding vouchers as forms of whole plants, voucher number 02-9-1 for ASR and voucher number 02-10-4 for AR, were deposited in the Center for Chinese Medicine, HKUST. Commercial DBT sample (Registration number HKC-07336, lot number 0SM4014) was bought from a local TCM store in Hong Kong.

### 2.2. Samples Preparation

The preparation of DBT under optimized conditions was described previously [1, 2]. In preparing DBT, AR and ASR in a weight ratio of 5 : 1 were boiled together in water according to the optimized conditions as described previously [1, 2]. Finally, the extracts were dried by lyophilization and stored at −20°C.

### 2.3. ^1^H-NMR Spectroscopy

One hundred mg of the dried extract was dissolved in 1 mL of water, from which 550 *μ*L was drawn and mixed with 50 *μ*L of a D_2_O solution with 0.2% TSP-d4 and 3 mM sodium azide. TSP-d4 acts as an internal standard for NMR, and sodium azide inhibits microbial growth. All particulate materials were removed by centrifugation at 13,000 × g for 1 min, and the supernatant was transferred to a standard 5 mm NMR tube. NMR spectra were acquired on a Bruker AV 400 MHz NMR spectrometer with a 5 mm PA BBO 400SB BBFO-H-D05 Z-gradient BB observe probe head, operating at 400.13 MHz ^1^H-NMR frequency at 298 K. Gradient shimming was used to improve the magnetic field homogeneity prior to all acquisition. ^1^H-NMR spectra of the samples were acquired using a 1D CPMG pulse sequence (RD-90°-t1-90°-tm-90°-acquire) to generate a spectrum with a reduced residual solvent peak. The experiment time for each sample was around 10 min. All spectra were Fourier-transformed, phase-corrected, and baseline-corrected manually.

### 2.4. Data Analysis

^1^H-NMR spectra obtained from each sample were phased, baseline-corrected, calibrated to the TSP internal standard resonance (*δ*_H_ = 0.00), and integrated using the rNMR [11]. The data were then formatted in XML for importing into MATLAB (version 2009b, The MathWorks, Natick, MA, USA) and SIMCA-P version 12.0 (Umetrics, Sweden). Each ^1^H-NMR spectrum was* Pareto*-scaled and divided into 1300 bins (bin width 0.0084 ppm). The summed intensity in each bin was used as a data point for principal component analysis (PCA). Discriminant analysis was used to pinpoint peaks in the spectra that were different between groups, which were then assigned using Chenomx Profiler, a module of Chenomx NMR Suite version 7.5, with input from online databases hmdb.ca [12], bmrb.wisc.edu [13], and the literature data [14]. The statistical significance of the changes in the integrated area of these assigned peaks was tested using a two-tailed Student's* t*-test.

## 3. Results

**Figure 1:**
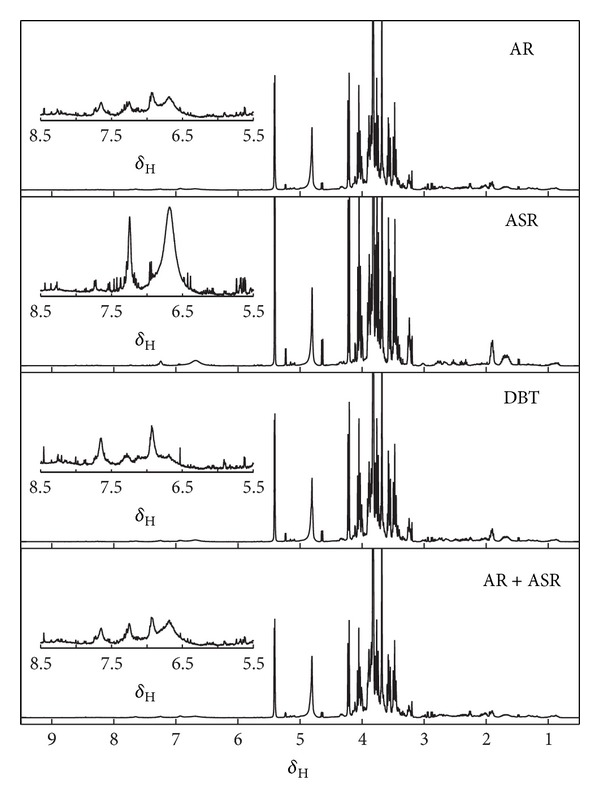
Average ^1^H-NMR spectra of four herbal extracts: AR, ASR, mixture of AR + ASR, and Danggui Buxue Tang (DBT). Spectra represented the mean of 5 replicates for AR, ASR, and DBT. For comparison, the spectrum AR + ASR was plotted by summing up spectra of AR and ASR in the ratio 5 : 1 to simulate the profile of a 5 : 1 mixture of AR extract and ASR extract. By visual inspection, the average ^1^H-NMR spectra of the DBT, AR, and AR + ASR showed a very similar profile but observable differences in the finer details. Abbreviations: AR: Astragali Radix; ASR: Angelica Sinensis Radix; DBT: Danggui Buxue Tang; AR + ASR: Astragali Radix + Angelica Sinensis Radix.

The mean ^1^H-NMR spectra of the replicates from the 3 groups of herbal extracts (AR, ASR, and DBT) were shown in Figure 1. For a better comparison, we summed up the AR and ASR spectra in a 5 : 1 ratio, so as to simulate the profile of a 5 : 1 mixture of AR extract and ASR extract, that is, AR + ASR. The difference between the profiles of AR and ASR was clearly visible. The profiles of AR, AR + ASR, and DBT showed a close similarity, likely because AR was the major component in the decoction. The contribution of ASR in the decoction could be recognized in a few peaks of DBT spectrum that were mainly in the sugar region (*δ*3.4–4).

**Figure 2:**
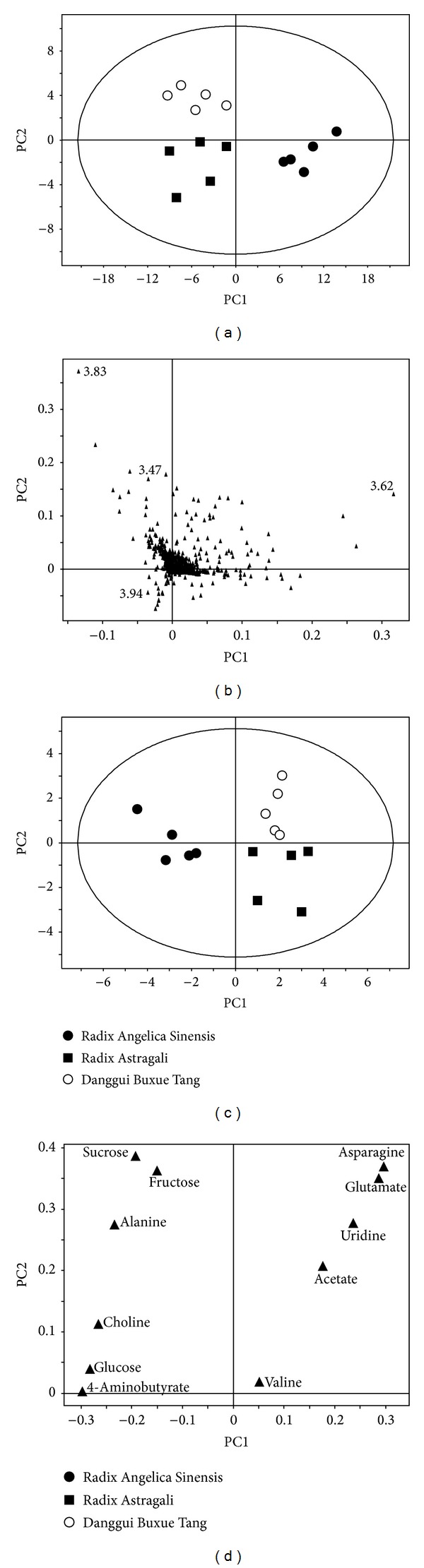
Chemometric analysis by principle component analysis (PCA). The score (a) and loading (b) plots from pattern recognition (PCA) of the whole spectra and the score (c) and loading (d) plots from pattern recognition (PCA) of targeted molecules. Overall, the score plot can successfully discriminate all 4 groups. Data are unit-variance scaled. Loading plot shows the molecules contributing to the separation (*n* = 5). Asparagine and glutamate are the markers that separate DBT from other samples.

In order to understand the similarity and dissimilarity among the samples, principle component analysis (PCA) was performed [15]. PCA score plot of the full spectra discriminated successfully all 3 groups of samples (Figures 2(a) and 2(b)). However, the corresponding loading plot, which is used to detect the variables responsible for clustering in the data, highlighted the differences in sugar region (*δ*3.4–4), because this region dominated the spectra. In order to understand how other metabolites contributed to the separation, we had chosen few known metabolites and reperformed the PCA data analysis. The PCA score plot of specific chemicals was very similar to the PCA score plot of the full spectra (Figure 2(c)). This result showed that the two profiling methods (based on whole spectra and selected chemical targets) produced similar discrimination. As expected, the AR cluster and the ASR cluster were well separated. Interestingly, the DBT data points were not found on the imaginary line drawn between the AR and the ASR clusters, as one might expect if DBT was merely a mixture of the two extracts. Rather, the DBT cluster was positioned far away in the upper-right corner, indicating that additional discriminatory features were present for the distinction of DBT.

**Figure 3:**
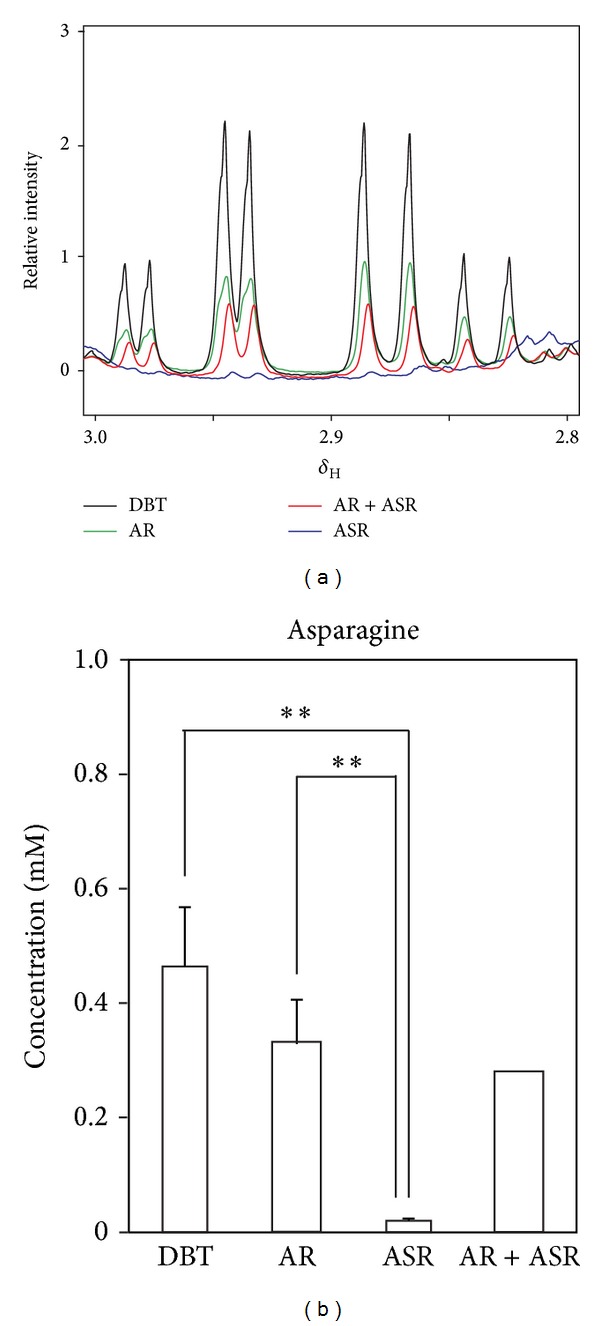
Quantification of asparagine in different extracts. Resonance of asparagine was indicated (a). The peak associated with asparagine in the ^1^H-NMR spectra was plotted and integrated to yield the concentrations in the extracts (b). DBT had the highest concentration of asparagine, followed by AR. Values are expressed as means ± SEM (*P ≤ 0.05, **P ≤ 0.01, and ***P ≤ 0.001), by Student's* t*-test. Abbreviations: AR: Astragali Radix; ASR: Angelica Sinensis Radix; DBT: Danggui Buxue Tang; AR + ASR: Astragali Radix + Angelica Sinensis Radix.

**Figure 4:**
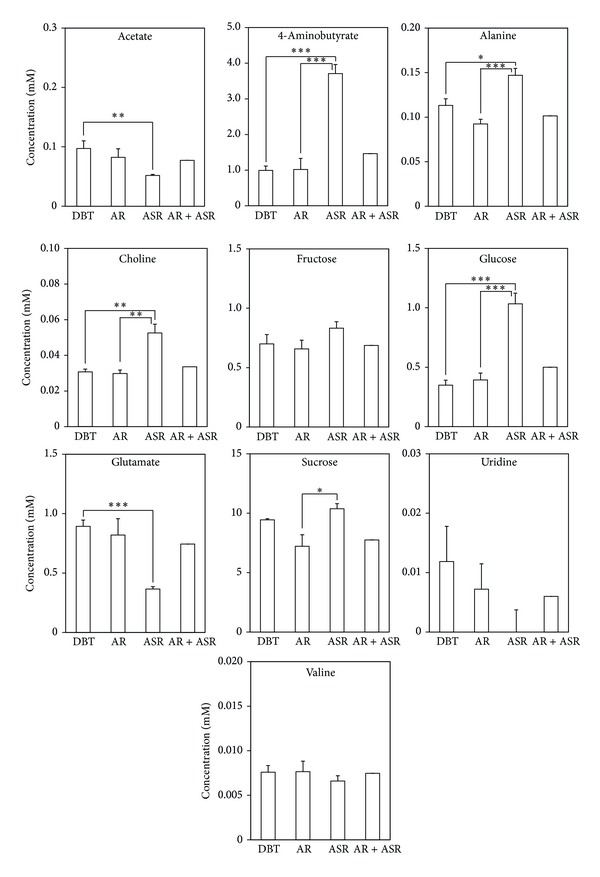
Quantification of identified metabolites in DBT and other herbal extracts. The peaks associated with identified metabolites in all of the ^1^H-NMR spectra were integrated to yield the concentrations in the extracts. Although glutamate was also observed to differentiate DBT samples from others, as in Figure 2(b), statistical analysis showed that the differences between DBT and AR groups were not significant. The values are expressed as means ± SEM (*P ≤ 0.05, **P ≤ 0.01, ***P ≤ 0.001), by Student's* t*-test. Abbreviations: AR: Astragali Radix; ASR: Angelica Sinensis Radix; DBT: Danggui Buxue Tang; AR + ASR: Astragali Radix + Angelica Sinensis Radix.

By studying the corresponding loading plots, we identified asparagine and glutamate as the potential metabolic markers that could separate DBT group from the other (Figure 2(d)).  Figure 3(a) shows the mean ^1^H-NMR spectra between 2.8 and 3.0 ppm, corresponding to asparagine. DBT had the highest concentration of asparagine, followed by AR. For comparison, we also showed the expected spectrum of a 5 : 1 mixture of AR extract and ASR extract. Statistical analysis showed that asparagine was higher in DBT group than that in AR and ASR groups (Figure 3(b)). The concentration of asparagine in DBT was approximately 2 times that of the value expected if DBT was a simple mixture of the water extracts of AR and ASR. Indeed, the amount of asparagine was minimal in ASR extract. The underlying reason for this phenomenon is unclear at present and will be a subject of a future study. Statistical analysis was also performed on all identified metabolites, which was shown in Figure 4. Although glutamate was also observed to differentiate DBT samples from other, as in Figure 2(d), statistical analysis showed that the differences between DBT and AR groups were not significant. In contrast, the difference between AR and ASR were clearly observed in other metabolites. For example, the levels of 4-aminobutyrate, alanine, choline, glucose, and sucrose were found higher in ASR group, as compared to AR group. Lastly, no uridine was observed at detectable level in ASR samples.

**Figure 5:**
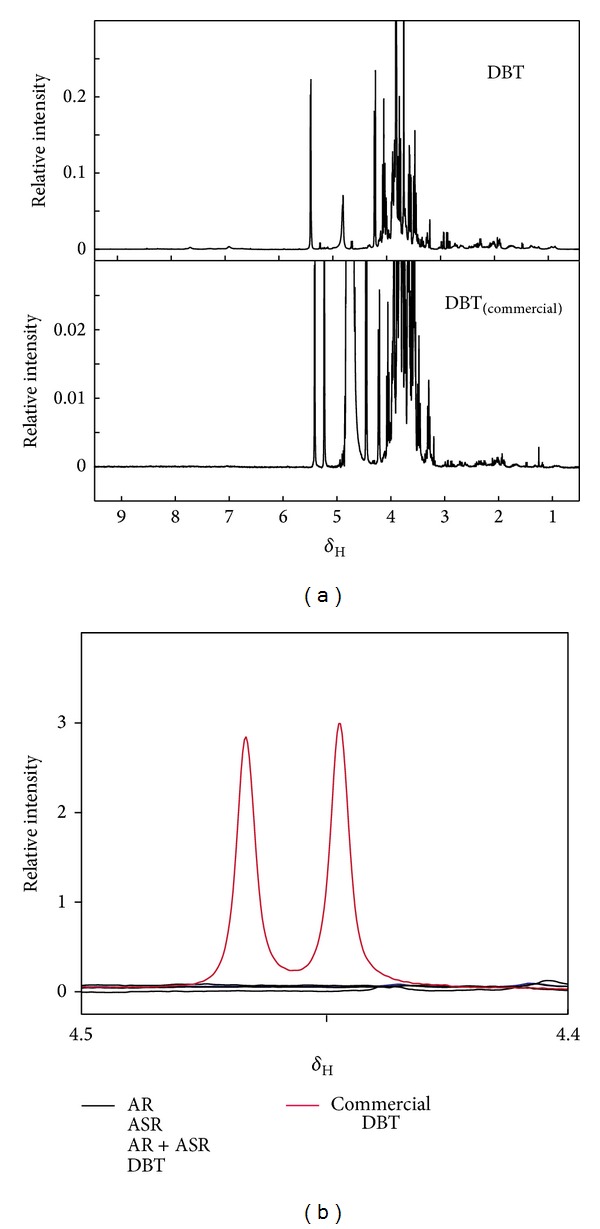
^1^H-NMR spectra of Danggui Buxue Tang and commercial Danggui Buxue Tang. (a)By visual inspection, DBT has overall higher peak intensity than commercial DBT. Overall features of both spectra are very similar. (b) Resonance of lactose was indicated. Commercial DBT is represented in red line, while DBT, AR, ASR, and AR + ASR are all represented in back lines. This shows that lactose is present in commercial DBT but not in any of our samples, including the raw materials.

We then proceeded to test a commercial preparation of DBT product using this method, aiming to confirm the quality and authenticity of this product. The product came in a powder form in 3.0 g net weight, with instruction to dissolve the powder in hot water without specifying the volume. We weighed an equal amount of this powder as our freeze-dried samples (100 mg), dissolved it in 1 mL water, and performed ^1^H-NMR metabolic profiling.  Figure 5(a) shows the overall spectra of both DBT prepared by our laboratory and commercial DBT. Overall, the features of the spectrum of the commercial DBT are similar to those of the DBT prepared in our laboratory. However, the overall intensity of commercial DBT was about an order of magnitude lower than that of the DBT prepared in our laboratory (note the difference in the scale of the *y*-axis). A strong doublet peak located at 4.45 ppm (Figure 5(b)) was observed in the commercial sample only. By searching the peak in Chenomx and in bmrb, we determined that the peak should originate from lactose.

## 4. Discussion

DBT is an ancient decoction made by boiling AR and ASR in 5 : 1 ratio. Dong et al. (2006) showed that the 5 : 1 ratio of AR and ASR should be the best ratio in both chemical compositions and biological efficacy [2]. They also showed that two herbs must be boiled together in order to achieve maximum activity. In this study, we had applied ^1^H-NMR metabolic profiling on 3 groups of samples: AR water extract, ASR water extract, and DBT decoction. We employed an identical extraction method, a condition mimicking the clinical preparation in TCM practice, to all three samples and analyzed an equal amount of dried extract in ^1^H-NMR.

Using chemometric methods, we compared raw ^1^H-NMR spectra and the profiles of identified metabolites. Principle component analysis (PCA) revealed that the profiles of the decoction DBT can be clearly distinguished from those of the individual herbs AR and ASR, although it is much more similar to the former than the latter. This is probably because AR is the major herb in the mixture. However, we observed that the DBT data points do not reside between the AR group and the ASR group, as one might expect, but rather form a distinct cluster away from the imaginary line between the AR and ASR groups. This indicates that DBT is not merely a proportional mixture of individual herbal extracts, and some additional features must be responsible for the discrimination. The PCA loading plot revealed that asparagine and glutamate, two common amino acid metabolites, are found at much higher concentrations than one expect for a mixture of AR and ASR. Indeed the concentrations of these two metabolites in DBT are higher than those in individual herbal extract.

Asparagine was not investigated in any previous study on the chemical composition of DBT. We speculated that this might be due to some unknown reactions or coextraction effects while boiling both herbs together to produce DBT. Although, asparagine is unlikely to be responsible for the biological activity of DBT, this finding from metabolic profiling indeed echoes the findings of Dong et al. (2006) [2], where they reported an elevated level of ferulic acid and a lowered level of Z-ligustilide in DBT, as compared to a postboiled mixture of AR and ASR extracts.

This finding has an important implication on the use of metabolic profiling for TCM quality control. At present, all available chromatographic or spectroscopic standards of TCM herbs, such as those in the Chinese Pharmacopoeia, are for pure herbal samples, often employing organic extraction procedures that are not relevant clinically. However, in practice, most of the TCM is consumed as water-based decoctions of multiple herbs, and most commercial ready-to-consume products are decoctions. Given their prevalence and popularity nowadays, one would argue that the quality control of such products is a more pressing concern and a greater challenge than that of whole herbs, which retain the outward appearance and genetic information for more traditional means of quality control. Given our and Dong et al.'s (2006) observation that a true decoction is not merely an additive mixture of the individual herbal extracts, an adequate quality control scheme for such TCM products would require standards of decoctions of authenticated herbs prepared in clearly specified, clinically relevant protocols [2]. This is currently an unmet need in the field.

As a demonstration of how ^1^H-NMR metabolic profiling can be used to assess the quality and authenticity of a commercial product, we tested a commercial powder-form sample sold over the counter in a TCM store in Hong Kong. According to the labeling, this product was extracted with 3.33 g of AR and 0.67 g of ASR, which adheres to the ancient DBT formula. There is no mentioning of the extraction procedure or other ingredients added. Our ^1^H-NMR metabolic profiling confirmed that the product was probably authentic, as the peak of the ^1^H-NMR spectrum matches well with that of the DBT decoction we prepared. However, the overall concentration appears to be about an order of magnitude lower. This is likely due to a large amount of lactose we observed in the commercial sample, as we compared the commercial sample to our dried DBT extract at the same weight. Lactose is a commonly used excipient in drug formulation as a volume filler to facilitate tablet compression [16].

As a method for quality control, ^1^H-NMR metabolic profiling is a faster method (~10 mins) compared to mass spectrometric (GC-MS or LC-MS) approach (~20 mins) [17, 18]. Also, a ^1^H-NMR metabolic profile of a biological sample could at once measure different metabolites in an unbiased manner, without the need to define, beforehand, the chemical markers to measure. Moreover, the intensity of ^1^H-NMR signal is directly proportional to the number of protons, enabling absolute quantification without pure standards. The limitation of ^1^H-NMR is that its sensitivity is lower (10 *μ*M), and is more suitable to measure primary metabolites than less abundant secondary metabolites, which are often used as chemical markers for TCM quality control, traditionally. This likely implies that ^1^H-NMR can play a complementary role in TCM quality control, in that it will monitor and quantify a different set of analytes than the typical TCM chemical markers. As we demonstrated in the analysis of the commercial sample, ^1^H-NMR can more readily detect contaminants, which will escape detection entirely in methods that rely on predefined chemical markers. In terms of ease of use, the data analysis and visualization of ^1^H-NMR metabolic profiling are also more involved, largely due to the complexity and richness of the data, but this is true of any profiling methods. The equipment and maintenance cost of ^1^H-NMR is also typically higher than conventional GC-MS and LC-MS platforms, but low-end bench-top NMR machines are now available which are comparably priced.

## 5. Conclusion

In conclusion, we applied ^1^H-NMR metabolic profiling for the quality control of a TCM decoction, for the first time. We successfully discriminated Danggui Buxue Tang (DBT), a simple but commonly used herbal decoction containing AR and ASR. As shown here and previously, ^1^H-NMR metabolic profiling is adequate in distinguishing closely related biological samples. For example, previous studies had shown that ^1^H-NMR metabolic profiling could readily distinguish species within the same genus and the same species grown in different regions, which should be a necessary task in quality control and not always possible with traditional methods [7, 8]. In this study, our data showed that ^1^H-NMR metabolic profiling could be used to distinguish the decoction from the water extracts of individual herbs, even if one of the herbs dominated the other. This suggests, as in the literature, that DBT is not merely a mixture of AR and ASR extracts, highlighting the need of generating profiles for decoction standards for quality control. We also demonstrated that this method could be used to verify the authenticity and quality of a commercial DBT product. By employing the clinically relevant extraction method of boiling the herbs in water, rather than the quicker methanol extraction typically used for this type of study in the laboratory, the metabolic profile we generated could be directly compared to that of a dried decoction product on the market. In the long term, a database of ^1^H-NMR metabolic profiles of TCM herbal decoctions would be highly desirable as a community resource for the quality control of TCM products, a pressing need for public health.

## Abbreviations

TCM: Traditional Chinese medicine
PCA: Principle component analysis
TSP-d4: Sodium 3-(trimethylsilyl) propionate-2,2,3,3-d4.
